# Effect of Deep Brain Stimulation on Speech Performance in Parkinson's Disease

**DOI:** 10.1155/2012/850596

**Published:** 2012-11-21

**Authors:** Sabine Skodda

**Affiliations:** Department of Neurology, Knappschaftskrankenhaus, Ruhr University Bochum, In der Schornau 23-25, 44892 Bochum, Germany

## Abstract

Deep brain stimulation (DBS) has been reported to be successful in relieving the core motor symptoms of Parkinson's disease (PD) and motor fluctuations in the more advanced stages of the disease. However, data on the effects of DBS on speech performance are inconsistent. While there are some series of patients documenting that speech function was relatively unaffected by DBS of the nucleus subthalamicus (STN), other investigators reported on improvements of distinct parameters of oral control and voice. Though, these ameliorations of single speech modalities were not always accompanied by an improvement of overall speech intelligibility. On the other hand, there are also indications for an induction of dysarthria as an adverse effect of STN-DBS occurring at least in some patients with PD. Since a deterioration of speech function has more often been observed under high stimulation amplitudes, this phenomenon has been ascribed to a spread of current-to-adjacent pathways which might also be the reason for the sporadic observation of an onset of dysarthria under DBS of other basal ganglia targets (e.g., globus pallidus internus/GPi or thalamus/Vim). The aim of this paper is to review and evaluate reports in the literature on the effects of DBS on speech function in PD.

## 1. Introduction

### 1.1. Dysarthria in Parkinson's Disease (PD)

Nearly 90% of individuals with Parkinson's disease (PD) develop voice and speech disorders (dysarthria) in the course of their disease [1]. Affected patients may complain about a quiet or weak voice and about difficulties to get speech started. Further, they often report that they are asked to repeat their words because listeners have difficulties to understand although patients themselves may self-estimate their speech as loud and sufficiently articulated [2]. Dysarthria can emerge at any stage of the disease and worsen in the later stages [3] causing a progressive loss of communication and leading to social isolation. Parkinsonian dysarthria has traditionally been interpreted as manifestation of rigor and hypokinesia on the speech effector organs [4] inducing to a multidimensional motor speech impairment including alterations of speech respiration, phonation, articulation, and prosody. Thus, based upon global clinical impression, hypokinetic dysarthria is characterized by a breathy and harsh voice, monotony of pitch and loudness, reduced stress, variable speech rate with short rushes of speech, and imprecise articulation resulting in a reduction of overall speech intelligibility [5–8]. From the therapeutic point of view, the effect of dopaminergic medication on different speech parameters and overall speech intelligibility in particular remains somewhat inconclusive. There are some reports of positive levodopa effects on tongue strength and endurance and of an improvement of speech intelligibility assessed by perceptual analysis in PD patients [9–11]. However, the majority of studies found no relevant effect of dopaminergic therapy on speech rate [12, 13], prosodic and phonatory parameters [14, 15], and overall intelligibility [16–18]. Nonpharmacological treatment strategies such as repetitive transcranial magnetic stimulation and laryngeal collagen augmentation injections seem to offer some positive, albeit transient, effects on voice and speech impairment in PD; however, the interpretation of data is limited by the very small number of so far treated patients [19, 20]. Up till now, behavioral speech therapy with special emphasis on rescaling the reduced amplitude of speech motor output is considered as the most effective therapeutic approach but is often found to be unsatisfying in a subgroup of patients [21, 22].

### 1.2. Effects of Lesional Surgical Treatment on Speech in PD

Before the rise of dopamine therapy, functional neurosurgery procedures, such as thalamotomy and pallidotomy, were used to treat symptoms of PD. Thalamotomy was generally performed in the ventrolateral and ventrointermediate nuclei of the thalamus to improve Parkinsonian tremor. Unilateral thalamotomy had been found to worsen speech independent if the lesion was in the dominant or nondominant hemisphere [23, 24]. However, there are also reports of neutral outcomes for speech following thalamotomy [25]. Bilateral thalamotomy had been associated with word blocking, slow speech and hypophonia, and a persistent worsening of dysarthria, some of the patients developed palilalia [26–28]. Because of these serious adverse events on Parkinsonian speech, bilateral thalamotomy has been abandoned for the treatment of PD.

Pallidotomy usually involved lesions of the posteroventral portion of the internal part of the globus pallidus and was used to alleviate Parkinsonian symptoms and reduce contralateral dyskinesias [29]. Concerning speech function, the majority of studies found no effect of pallidotomy on hypokinetic dysarthria [30–33]. Though, some studies describe positive changes of labial force production and stability in a subgroup of patients [34] and an improvement of phonatory and articulatory measurements in PD speakers after unilateral and bilateral pallidotomy [35, 36]. On the other hand, others report on a worsening of speech function with development of transient dysarthria, facial weakness, swallowing problems, and alterations in verbal fluency [37]. In summary, the current literature about the effects of ablative surgical procedures on motor speech function in PD remains equivocal; investigations conducted in the early stereotactic era at least suggested that least bilateral thalamotomy was most likely to result in negative speech outcomes [38].

### 1.3. Effects of Deep Brain Stimulation on Speech in PD

In the last years, numerous studies have proven the beneficial effects of high-frequency deep brain stimulation (DBS) of the subthalamic nucleus (STN), globus pallidus internus (GPi), and the ventral intermediate nucleus of the thalamus (Vim) on motor symptoms in PD [39–43]. However, the effects of DBS on voice and speech have been found to be variable or even adverse. According to the speech item of the Unified Parkinson's Disease Rating Scale (UPDRS), the prevalence of dysarthria under STN-DBS has been reported to vary between 1% after 6 months up to 70% at three-year followup with an average of 9.3% [44–46]. On the other hand, there are also reports of an amelioration of distinctive parameters of voice and nonspeech articulatory measures in individual PD patients under STN-DBS (e.g., [47]). In a similar vein, studies on speech performance under DBS of targets different from STN have produced heterogeneous results. Under GPi-DBS, overall speech performance based upon perceptual rating showed an improvement in a small series of seven patients [48], whereas worsening of speech intelligibility has been observed in other studies [49–51]. Similarly, Vim-DBS has been reported to have a worsening effect on perceptual assessment and electrophysiological outcome parameters of speech in patients with tremor-dominant PD as well as in patients treated with Vim-DBS for essential tremor [52–56].

Based upon these observations, growing interest has been focused on the impact of DBS on speech in PD, and numerous subsequent investigations with more subtle analyses of overall speech performance and of distinctive speech parameters have been conducted to gain a better understanding of the mechanisms why and how DBS can induce alterations of voice and speech in some PD patients.

The aim of the current paper is to review and discuss the existing studies on voice and speech performance in PD as a basis for a better information and management of patients.

## 2. Methods

A Medline literature search were undertaken including articles published until September 2012 using the search terms “Parkinson's disease/PD” and “deep brain stimulation/DBS” and “dysarthria” and/or “speech” and/or “voice”. The search results were narrowed down to investigations focused on voice and speech performance under DBS based upon qualitative description or perceptual, acoustic or electrophysiological analyses, since it has been noticed that the UPDRS speech item alone shows poor sensitivity to detect speech problems [57]. Furthermore, the reference lists of the chosen articles were checked for additional publications fulfilling these criteria.

## 3. Results

A number of 35 publications were identified with numbers of participants ranging from one (case reports) up to 57 ([56, 58–91], see Table 1). The great majority of data (*n* = 34) were derived from patients under STN-DBS; three studies compared speech performance under STN-DBS with DBS of the caudal zona incerta (cZi), and there were three further investigations on STN-GPi and STN-Vim. Concerning methods, studies differed considerably with respect to the participants' characteristics (disease duration, dosage of concomitant medication, time period after DBS surgery, etc.), underlying speech tasks (sustained phonation, syllable production, word or sentence reading, free monologue, and performance of nonspeech movements of the articulatory muscles), and the kind of analysis (extensive perceptual assessments, acoustic analysis of different sets of speech parameters, and electrophysiological measurements of articulatory and phonatory function) which limits the direct comparability.

Therefore, the main findings of the studies are described and discussed in the following sections.

### 3.1. Impact on STN-DBS on Voice and Speech

Since over the last decade, the favoured DBS target for the treatment of motor symptoms in PD was the STN, most data about a possible deterioration of speech performance are derived from patients under STN-DBS. According to UPDRS speech item ratings alone, a meta-analysis of 37 cohorts comprised of 921 patients reported an incidence of dysarthria as adverse event under STN-DBS of 9.3% [46] which is in general confirmed by other small studies (e.g., [64]). On the other hand, beneficial effects on speech performance are documented at least in individual patients although the improvement was much less pronounced than that on limb movements and tended to decrease in the long term (e.g., [82, 92, 93]). There are further studies which combine perceptual assessment of overall speech function with acoustic analysis and electrophysiological measurements which suggested that STN-DBS can improve articulatory and phonatory components such as loudness in Parkinsonian speech [77, 78, 85, 87, 88, 90, 91, 94]. For example, in one investigation, the authors found an improvement of articulatory force and overall speech function in the majority of 26 PD patients with STN-DBS using perceptual analysis and electrophysiological measurements [85]. In another study, no negative effects of STN-DBS were seen in 12 PD patients; on the contrary, some aspects of speech as vocal tremor tended to improve but without effects on global speech intelligibility [78]. Worsened overall speech performance according to perceptual ratings was seen in another study on 19 patients under STN-DBS; however, technical measures showed stimulation-induced improvements of single speech dimensions affected by the PD-specific motor disorder [77]. The authors concluded that STN-DBS could reduce designated disease-inherent dysarthrophonic symptoms, such as reduced loudness or glottic tremor, however, that these actions on speech could be outweighed by a general dysarthrogenic effects of STN-DBS, probably based on a decline of complex (e.g., prosodic) functions [77]. Similarly, other investigators proposed that STN-DBS has a differential impact on different modalities of Parkinsonian speech with the potential to ameliorate phonation, however, at the cost of a deterioration of articulatory capacities leading to a reduced overall speech intelligibility [69, 79, 94]. Furthermore, STN-DBS was reported to induce abnormalities in speed and regularity of nonspeech syllable repetition as a possible hint for a negative effect on basal motor speech performance [63].

### 3.2. Influence of Stimulation Parameters and Side of STN Stimulation

Other investigators intended to find correlations between parameter settings such as amplitude, frequencies, and polarity between the stimulation electrode contacts and speech performance [64, 70, 74, 75, 80, 95, 96]. Consistently, the authors reported a deterioration of dysarthria rated by perceptual assessments under high-amplitude or high-frequency stimulation settings which, however, were required for the optimization of motor performance at least in some individual patients. Likewise, the exact contact position within the STN was found to be of importance since stimulation right within the STN and especially in the medial and/or posterior portion of the nucleus was linked with poorer speech intelligibility [70, 74, 75]. In another investigation on 57 PD patients the exact positions of the STN electrodes were correlated to clinical outcomes with the result that better symptom relief with reduced need for post-op medication was expected in patients whose electrodes were accurately positioned in both STN [64]. However, even in the subgroup of 36 patients with exact electrode position in both STNs, 36% showed a deterioration of speech under stimulation compared to 50% featuring an improvement [64]. Two other studies surveyed a possible differential impact of left- and right-sided STN stimulation on different aspects of speech performance and found that selective left-sided stimulation had a profoundly negative effect on prosody, articulation, and hence, intelligibility [95, 96]. 

### 3.3. Impact of Presurgery Speech Performance and Microlesion Effects

Up till now, there are only few investigations with speech testings before and at certain time intervals after DBS surgery which would be a reasonable approach to define subgroups of PD patients who are particularly on risk to experience deterioration of speech under STN-DBS or to identify a possible microlesion effect of electrode placement [58, 60, 65, 70, 83, 88, 90]. In the largest of these studies, 32 PD patients under STN-DBS were tested pre- and postsurgically with several follow-up examinations and compared with a group of medically treated PD patients [70]. Dysarthria was rated perceptually according to the widely used assessment for the Intelligibility of Dysarthric Speech battery. As a main result, speech intelligibility deteriorated on average by 14% after 1 year of STN-DBS when the patients were off-medication/on-stimulation and by 13% in the off-medication/off-stimulation state compared to off-medication state preoperatively. Similar results were found in the on-medication/on-stimulation state when compared to the on-medication state preoperatively (average deterioration of 17%). However, there was a substantial variability between individual patients, even with an improvement of dysarthria in 7 patients. In the medical treatment group, the decline of speech intelligibility after 1 year lays within a comparable range. The authors found a correlation of poorer speech outcome after 1 year and a higher presurgical general motor impairment in the on-medication condition, probably explained by the presence of nondopaminergic pathology. Furthermore, high voltage stimulation of medially located electrodes on the left STN was found to be associated with a significantly higher risk of speech deterioration [70]. Another study on 7 patients found no consistent effects of DBS surgery alone (i.e., no hint for microlesion effect) and no consistent stimulation effect on speech under STN-DBS after three months but a slight improvement of pitch variability and sound pressure levels under stimulation six month post-op [88]. Another two studies with PD patients tested before and 12 months after DBS surgery in the stimulation off-condition provided evidence for a progressive reduction of phonatory control, but not of speech intensity, which was interpreted as either progression of the disease, an effect of reduced post-op levodopa dosage, or a microlesion effect [58, 60].

### 3.4. Summary

#### 3.4.1. Impact on STN-DBS on Speech and Possible Mechanisms

As a first recapitulation of these data, the impact of STN-DBS on speech performance can be variable, and the available data still do not allow predicting the risk of the onset or deterioration of dysarthria in the individual patient. STN-DBS seems to have some potentials to ameliorate at least phonatory dysfunctions as voice tremor and reduced loudness; however, these beneficial effects might be counterbalanced by a prodysarthrogenic actions whose mechanisms are not yet fully understood. Since several studies document an association of dysarthria with higher voltage/frequency STN stimulation, one might assume a spread of current to the corticobulbar pathways for laryngeal motor control with an induction of a spastic/pseudobulbar dysarthria [93]. However, this proposed mechanism should not only induce a deterioration of connected speech performance, but also of other vocalizations as sustained phonation which has not been found in the previous investigations (e.g., [63, 75]). Current spread into other pathways, namely, the pallid fugal and cerebellothalamic fibers seem to more adequately account for speech impairment, especially in patients with electrodes placed within the medial portion of the STN [97]. Furthermore, the optimal implantation position of the electrodes is typically chosen on the basis of limb motor effects of stimulation disregarding the possibility that STN could have a different role or somatotopy for speech and body motor control. This assumption is corroborated by a positron emission study on PD patients which could demonstrate different patterns of activation with speech production and hand movements which were differentially modulated by STN-DBS [82]. Additionally, one might assume a further microlesional effect induced by the electrode insertion itself which could induce an earlier decompensation of the already dysfunctional speech system in the course of PD [58, 70]. This hypothesis would at least account for the finding that PD patients with higher presurgical global motor impairment are on higher risk to develop speech problems within the first year under STN-DBS, even in the off-stimulation condition [70]. Another possible factor could be the reduction of dopaminergic medication under STN-DBS since one could assume that a certain amount of medication could still be required to ensure a satisfying speech performance. However, although the available data are somewhat inconsistent, dopaminergic medication has been estimated to have at best limited effects on speech performance, and deterioration of dysarthria in the course of PD rather seems to reflect nondopaminergic dysfunction [98]. Therefore, it is not likely that a deficit of dopaminergic medication relevantly accounts for the observed speech abnormalities under STN-DBS, the more so, since indeed for some patients, speech was reported to be worse on-medication/on-stimulation [70, 93, 99].

Summarized, since there are no established algorithms for the prediction of the impact of STN-DBS on speech for the individual patient uptill now, neurologists have to carefully keep in mind the possibility of speech deterioration and to inform the patient accordingly, when the indication of STN-DBS is discussed. Patients who are suffering from symptoms unresponsive to dopaminergic therapy (with higher motor impairment in the best medication on state) should be aware of the possibility of a detrimental effect of STN-DBS on speech, and a preexisting severe Parkinsonian dysarthria cannot be the main indication for STN-DBS.

In the postsurgical management of PD patients under STN-DBS, patients should be carefully monitored concerning speech function. If speech performance shows a relevant deterioration in the on-stimulation condition, a meticulous testing and adjustment of electrode contact sides and stimulation parameters can be helpful to achieve a clinically optimal balance between satisfactory motor function and intelligibility of speech in the individual patient. In some cases, however, it can be necessary that the patient himself can vary the stimulation parameters within a certain preset range, for example, reduce the stimulation amplitude for a better speech performance during longer conversations. If these strategies remain disappointing, speech therapy should be provided betimes, wherein best evidence has been documented for Lee Silverman Voice Treatment (LSVT) which has shown to be effective at least in a subgroup of patients with impaired speech intelligibility under STN-DBS [100, 101].

#### 3.4.2. Impact of DBS of GPi and cZi on Speech

In contrast, effects of DBS of the GPi on speech performance have only scarcely investigated so far. In one study including 27 PD patients velocity of externally scaled jaw movements was found to be significantly reduced under STN-DBS, but not under GPi-DBS, leading to the authors' recommendation to consider the GPi at the preferable target for PD patients with preexisting oromandibular dysfunction [65]. In large controlled trials, speech performance has mostly been assessed by item 18 of the UPDRS Motor Scale, which shows poor sensitivity to detecting speech problems and indeed identified only 38% of patients with speech deterioration in one study [57, 70]. At least according to the UPDRS speech item, the rate of dysarthria as an adverse event seems to occur less often under GPi-DBS than under STN-DBS [42, 102, 103].

Recently, DBS of the caudal zona incerta (cZi) has been compared concerning its effect on speech performance in comparison to STN-DBS in small groups consisting of 7 to 8 patients [58, 60, 61]. Results showed a differential impact of cZi-STN on different measures of speech, whereas phonatory control remained unaffected by cZi-STN (and STN-DBS), patients showed a small but significant reduction of speech intensity and a decrease in articulation rate and quality [58, 60, 61].

The ventral intermediate nucleus of the thalamus (Vim) is an established target for the treatment of medically intractable tremor syndromes of different etiologies. Since Vim-DBS cannot alleviate the other motor manifestations of PD, thalamic stimulation is only used as an individual option in exceptional tremor-dominant PD cases. Therefore, no systematic studies on the impact of Vim-DBS on Parkinsonian speech are available. However, there are reports on an induction of dysarthria under Vim-STN interpreted as being induced by the spread of current-into-adjacent pathways [52, 104, 105].

## 4. Conclusion

DBS has been proven to be an effective treatment for PD patients with refractory tremor or motor fluctuations, but its impact on speech can be variable, and deterioration of speech intelligibility can counterbalance the motor benefits of the procedure. Uptill now, the mechanisms responsible for a worsening of Parkinsonian dysarthria under STN-DBS are not fully understood, but it is plausible to assume a combination of preexisting hypokinetic dysarthria as a manifestation of progressive and nondopaminergic dysfunction with microlesion- and stimulation-induced effects as spreading of current-into-adjacent pathways. According to very few and preliminary data, speech function seems to be less compromised under GPi-DBS than under STN-DBS, but this first impression demands further corroboration. Consecutive studies with large numbers of patients are warranted which refine and further develop the previous investigations (e.g., [70, 85, 106]) including patients at presurgical and defined follow-up intervals under DBS with subtle speech investigations (ideally consisting of perceptual ratings of overall speech intelligibility in combination with objective acoustic analysis and/or electrophysiological testings) in on- and off-stimulation conditions. The aim of these studies should be to gain a better understanding of the underlying pathophysiology to identify patients who are at risk to develop speech deterioration under STN-DBS. Furthermore, investigations on the effects of GPi- and cZi-STN on speech performance are necessary to decide which target is most appropriate in the individual PD patient for best motor and speech performance. 

## Figures and Tables

**Table 1:** 

Sample size	Target (all studies with stimulation on versus stimulation off)	Outcome measure	Results	R
*n* = 16	STN (*n* = 8), cZi (*n* = 8); pre-op and 12 months post-op	Phonatory control: alternations between voicing and voiceless states in a reading task	Progressive deterioration of phonatory control but unaffected by DBS	[58]

*n* = 6	STN	Perceptual ratings and acoustic analysis	Deterioration of overall speech performance (perceptual ratings); mixed results concerning single speech parameters (acoustic analysis)	[59]

*n* = 16	STN (*n* = 8), cZi (*n* = 8); pre-op and 12 months post-op	Mean intensity during reading, intensity decay during syllable repetition	STN: increase of intensity; cZi: slight reduction of intensity during reading; no significant change of intensity decay	[60]

*n* = 14	STN (*n* = 7), cZi (*n* = 7)	Articulatory capacity, accuracy of plosive consonants	STN: increased articulatory rate; cZi: deterioration of articulatory rate and quality	[61]

*n* = 10	STN	Acoustic analysis of different speech parameters	Mixed results with positive and negative effects on single speech parameters	[62]

*n* = 14	STN	Acoustic analysis of syllable repetition precision	Deterioration of syllable repetition capacity	[63]

*n* = 57	(I) *n* = 36 with both electrodes within STN, (II) *n* = 16 with only one electrode within STN, (III) *n* = 5 with no electrode within STN	Perceptual rating according to UPDRS speech item	(I) 50% improvement, 36% deterioration of speech; (II) 69% improvement, 25% deterioration (III) 100% deterioration	[64]

*n* = 27	STN and GPi; pre-op and 6 months post-op	Peak velocities of jaw movements	STN: deterioration of jaw movement velocity GPi: no deterioration	[65]

*n* = 11	STN; stimulation frequencies with 60 Hz and 130 Hz	Perceptual rating, acoustic analysis, and aerodynamic measurements	Amelioration of outcome measures under 60 Hz stimulation	[66]

*n* = 2	STN	Case reports: description of speech performance	Reoccurrence of severe stuttering	[67]

*n* = 11	STN	Acoustic analysis of different speech tasks	No changes in speech performance	[68]

*n* = 2	STN	Articulatory accuracy measured by electropalatography	Deterioration in one patient, amelioration in the other patient	[69]

*n* = 32	STN; pre-op, 1 month, 6 months, and 12 months post-op	Perceptual analysis based upon validated rating scale	Amelioration of speech in *n* = 7; deterioration in the other patients by an average of 14.2% ± 20.15% off-medication; deterioration more often with high voltage and medially located electrodes within the left STN	[70]

*n* = 17	STN	Aerodynamic measures	Increased intraoral pressure in *n* = 7; increased velopharyngeal closure in *n* = 5	[71]

*n* = 9	STN	Perceptual and acoustic analysis	Amelioration of voice, no influence on fluency	[72]

*n* = 18	STN	Aerodynamic measures	Increased inspiratory driving pressure (*n* = 9); increased vocal fold closure (*n* = 9); more benefit from low-frequency stimulation	[73]

*n* = 10	STN; 4 V (high) and 2 V (low) voltage	Perceptual analysis based upon validated rating scale	Deterioration during high-amplitude stimulation in *n* = 6, in patients with electrodes in medial and/or posterior position	[74]

*n* = 14	within and above STN; 4 V (high) and 2 V (low) voltage	Perceptual analysis based upon validated rating scale	Deterioration of speech during high-amplitude stimulation independently from side of stimulation	[75]

*n* = 19	STN	Acoustic analysis of sustained vowel phonation	Improvement of single measures of voice	[76]

*n* = 19	STN	Perceptual ratings by patient, physician, and professional speech therapist, additional acoustic analysis	Deterioration of overall speech performance (perceptual ratings); amelioration of single speech/voice parameters (acoustic analysis)	[77]

*n* = 12	STN	Perceptual rating and acoustic analysis	No deterioration; amelioration of glottal stability and vocal tremor	[78]

*n* = 9	STN	Acoustic analysis of articulatory and phonatory function	Mixed results with improvement or deterioration of articulation and phonation in different patients	[79]

*n* = 10	STN, different parameter settings	Perceptual analysis of different speech tasks	Deterioration in *n* = 4 patients under “normal” stimulation parameters; further deterioration during high-amplitude and/or frequency stimulation	[80]

*n* = 4	STN	Qualitative description and acoustic analysis	Mixed results with improvement and deterioration of speech in the different patients	[81]

*n* = 10	STN	Perceptual rating according to UPDRS speech item, PET study	Improvement of overall speech performance	[82]

*n* = 7	STN; pre-op and 3 months post-op	Perceptual dysarthria assessment and rating according to UPDRS speech item	Modest improvement of lip movements, loudness, and pitch; slight reduction of intelligibility	[83]

*n* = 16	STN	Acoustic analysis and force measurements of articulatory muscles	Decrease of reaction and movement time of articulatory organs; increase of maximal strength and precision; improvement of respiratory and phonatory function	[84]

*n* = 26	STN; follow up at several years post-op	Perceptual ratings, measurement of articulatory force (lip and tongue force)	Improvement of articulatory force; deterioration of overall speech performance (perceptually rated) in a subgroup of patients	[85]

*n* = 1	STN	Case report: descriptive	Emergence of dysfluencies under STN-DBS	[86]

*n* = 26	STN	Acoustic analysis	Improvement of distinctive speech parameters	[87]

*n* = 7	STN; pre-op and 3 months and 6 months post-op	Acoustic analysis	Mild improvement of sound pressure level and pitch variability	[88]

*n* = 14	STN (*n* = 7), Vim (*n* = 7)	Measurement of articulatory force (lip and tongue force)	Vim: deterioration of static and dynamic control of articulatory organs STN: improvement	[89]

*n* = 1	STN, pre-op and 2 years post-op	Descriptive	Improvement of oral control and intelligibility	[90]

*n* = 10	STN	Perceptual rating according to UPDRS speech item, measurement of articulatory force (lip and tongue force)	Improvement of static and dynamic control of articulatory organs; improvement of reaction time; improvement of overall speech performance	[91]

*n* = 23	Vim (patients with tremor)	Perceptual rating	Development of dysarthria in *n* = 7	[52]

STN: Subthalamic nucleus, GPi: Globus pallidus internus, cZi: Caudal zona incerta, R: Reference.
